# Dietary intake and phthalates body burden in boys and girls

**DOI:** 10.1186/2049-3258-73-5

**Published:** 2015-02-09

**Authors:** Qing Shen, Huijing Shi, Yunhui Zhang, Yang Cao

**Affiliations:** Unit of Biostatistics, Division of Epidemiology, Institute of Environmental Medicine, Karolinska Institutet, Stockholm, 17177 Sweden; School of Public Health, Fudan University / Key Laboratory of Public Health Safety, Chinese Ministry of Education, Shanghai, 200032 China

**Keywords:** Dietary intake, Phthalate metabolites, Body burden, Schoolchildren

## Abstract

**Background:**

Phthalates are a group of environmental endocrine disruptors and have been ubiquitously applied in industrial field. Few studies had investigated how dietary intake was related with phthalate body burden in children.

To determine the relationship between phthalate body burden and dietary intake among school age children in Shanghai, China.

**Methods:**

Four hundred and thirty schoolchildren aged 8–16 years were recruited in a cross-sectional study with 18 months follow-up in Shanghai, China during 2010–2012. Data of questionnaire-based dietary intake were collected and urinary phthalate concentrations were measured. Associations between frequency of dietary intake and phthalate metabolite concentrations were evaluated by stepwise multiple linear regression model.

**Results:**

Positive association was found between mono-butyl phthalate and seafood, and negative associations were found between mono-butyl phthalate and dried fruits and vegetables. Egg consumption showed negative association with all di-(2-ethylhexyl) phthalate-related metabolites.

**Conclusions:**

Some food types were identified to be associated with phthalate body burden and diet might be a source of phthalate exposure among Chinese schoolchildren.

## Background

Phthalates are a group of environmental endocrine disruptors and have been ubiquitously applied in industrial field. They are incorporated into plastics for flexibility and durability as plasticizers [1], and can leach out during the usage of daily products. Children are more susceptible towards these toxic substances during the time windows of physical and reproductive development, since phthalates can imitate the role of biosynthetic matters and interfere with regulation of sex hormones that may lead to potential adverse health outcomes such as precocity and testicular dysgenesis syndrome [2, 3]. Recent studies also revealed the associations between phthalate exposure and higher blood pressure, increased insulin resistance, obesity, asthma and airway diseases in childhood [4–7]. Dietary intake has been noted as a significant route of phthalate exposure, and several daily food were associated with phthalate body burden in some countries [8–15]. For example, high concentrations of phthalates, especially di-(2-ethylhexyl) phthalate (DEHP), were found in daily food such as cream, fish, carcass meat, eggs and poultry [16–18]. Studies in the United States indicated that DEHP and high-molecular-weight phthalate metabolites were positively associated with the consumption of poultry and meat-related food, while the metabolites of di-ethyl phthalate (DEP) were related to vegetable consumption [9, 19]. However, in developing countries where higher risk of exposure to foods contaminated by phthalates might exist, studies are still scarce in investigating how dietary intake relates with phthalate body burden in vulnerable group such as children in quantified ways. Considering the wide range of phthalate contamination in daily food and the higher risk of exposure to phthalates for children compared to for adults, this study intended to assess the phthalate body burden through dietary intake focusing on school-age children in China.

## Methods

The study is a cross-sectional study among schoolchildren aged 8–16 years in Shanghai, China in 2012, aiming to determine the relationship between questionnaire-based dietary intake and urinary phthalate metabolite levels. In total, 430 students (222 boys, 208 girls) were recruited for anthropometric measures, dietary intake survey and laboratory tests.

Anthropometric measures, including body weight and standing height, were assessed by trained health workers. Data on demographics, parental information and dietary intake within last three months were collected using Food Frequency Questionnaire (FFQ). The FFQ used was validated in Chinese population for assessing health outcomes and food consumption [20]. The questionnaire included four major food categories (primary plant food, primary animal food, processed plant food and processed animal food) and 25 subgroups of food items, especially the frequently consumed food items that the Chinese children commonly consumed. Other dietary information of whether having the frequently-reported types of food containing estrogen during critical growth periods was also included. For every food group, the respondents may choose the consumption frequency from never, 1–3 times per month, 1–2 times per week, 3–4 times per week, 5–6 times per week and every day, based on their own consumption. And scores 0, 1, 2, 3, 4 and 5 were assigned to represented the corresponding frequencies. The total score was developed to quantify the total consumption of four major food categories. Spot urinary samples were collected on the day of physical examination and stored in phthalate-free container, and transferred on dry ice to the Key Lab of Public Health Safety of the Ministry of Education of China at Fudan University, Shanghai. The concentrations of six phthalate metabolites in urine were analyzed, including mono-butyl phthalate (MBP), mono-methyl phthalate (MMP), mono-ethyl phthalate (MEP), mono (2-ethylhexyl) phthalate (MEHP), mono (2-ethyl-5-hydroxyhexyl) phthalate (MEHHP) and mono (2-ethyl-5-oxohexyl) phthalate (MEOHP). Concentrations of MEHP, MEHHP and MEOHP were summed up as total concentration ΣMEHP in μg/L since they shared the same parental phthalate of DEHP. The enzymatic deconjugation of the glucuronidated metabolites, solid-phase extraction, separation were resolved by an Agilent 1100 Series high-performance liquid chromatography (HPLC) system (Agilent Technologies, Santa Clara, CA) and detected by an API 2000 electrospray triple quadrupole mass spectrometer (ESI-MS/MS; Applied Biosystems, Foster City, CA). Levels below the limit of detection (LOD) were set by dividing the LOD by two. Analyses on concentrations of phthalate metabolites were performed on specific gravity-corrected basis recommended by Hauser et al. (Atago PAL10-S, Tokyo, Japan) [21]. Associations between frequencies of dietary intake and phthalate metabolite concentrations were evaluated by the stepwise multiple linear regression model. Because all the distributions of phthalate metabolites were highly-skewed, the analyses were performed on log-transformed basis.

## Results

The summary of specific-gravity adjusted urinary concentrations of phthalate metabolites were shown in Table 1. MBP had the highest geometric mean of 21.88 μg/L, while MEHP had the lowest geometric mean of 0.98 μg/L. No significant difference was found between boys and girls.

**Table 1:** **Geometric mean (95% confidence interval) of specific-gravity adjusted urine phthalate metabolites levels (μg/L)**

	Boys (n = 222)	Girls (n = 208)	All (n = 430)
**MBP**	22.32 (19.47, 25.59)	21.41 (18.79, 24.39)	21.88 (19.91, 24.04)
**MMP**	15.56 (13.73, 17.65)	15.77 (14.26, 17.44)	15.67 (14.45, 16.98)
**MEP**	4.25 (3.58, 5.06)	4.01 (3.40, 4.74)	4.14 (3.67, 4.66)
**MEHP**	0.96 (0.82, 1.11)	1.01 (0.87, 1.19)	0.98 (0.88, 1.10)
**MEHHP**	9.22 (8.13, 10.45)	9.41 (8.16, 10.84)	9.31 (8.47, 10.23)
**MEOHP**	3.44 (3.05, 3.88)	3.60 (3.18, 4.07)	3.51 (3.22, 3.83)
**∑MEHP** ^**a**^	14.01 (12.39, 15.83)	14.57 (12.78, 16.62)	14.28 (13.06, 15.61)

^a^Sum of MEHP, MEHHP and MEOHP, as DEHP-related metabolites.

The coefficients in multiple linear regression model adjusted for age, gender, body mass index (BMI), parents’ average education level, parents’ smoking and alcohol consumption and other types of food intake denoted the change of log-transformed specific-gravity adjusted phthalate metabolite level with per unit increase in food frequency score. The statistically significant results were shown in Table 2. Specifically, sea food presented significantly positive association with MBP level (b = 0.089, p = 0.048), while dried fruits and vegetables showed negative association with MBP level (b = -0.130, p = 0.007). Light color vegetables had significantly negative association with MMP levels (b = -0.124, p = 0.001), and luncheon meats and processed meats had positive association with the concentration of MMP (b = 0.116, p = 0.011). MEP level only showed significantly negative association with preserved food (b = -0.161, p = 0.037). In addition, eggs showed negative association and freshwater fish had positive association, respectively, with all di-(2-ethylhexyl) phthalate (DEHP)-related metabolites (MEHP, MEHHP, MEOHP) and their sum level (∑MEHP) (see Table 2). The negative associations of light color vegetables and lactic acid beverages, and the positive association of fresh fruits and freshwater fishes were found with secondary DEHP metabolites (MEHHP and MEOHP) and ∑MEHP levels. Besides, soft drinks showed positive associations with MEOHP and ∑MEHP levels (b = 0.098, p = 0.009; b = 0.088, p = 0.023, respectively).

**Table 2:** **Linear regression analyses of log-transformed specific-gravity adjusted phthalate metabolites and intake frequency of daily foods**

Phthalate metabolites	Food types	Crude analysis		Adjusted analysis ^a^	
		B (95% CI)	p-value	B (95% CI)	p-value
**MBP**	-Sea food	0.105 (0.025 to 0.185)	0.010*	0.089 (0.001 to 0.176)	0.048*
	-Dried fruits and vegetables	0.061 (-0.019 to 0.142)	0.135	-0.130 (-0.224 to -0.036)	0.007*
**MMP**	-Light color vegetable	-0.030 (-0.096 to 0.036)	0.365	-0.124 (-0.120 to -0.048)	0.001*
	-Luncheon meats or processed meats	0.100 (0.030 to 0.169)	0.005*	0.116 (0.027 to 0.205)	0.011*
	-Lactic acid beverages	-0.002 (-0.056 to 0.051)	0.928	-0.065 (-0.125 to -0.004)	0.038*
**MEP**	-Preserved food	-0.130 (-0.244 to -0.015)	0.027*	-0.161 (-0.313 to -0.010)	0.037*
**MEHP**	-Eggs	-0.100 (-0.187 to -0.014)	0.023*	-0.143 (-0.237 to -0.048)	0.003*
	-Freshwater fish	0.081 (-0.009 to 0.172)	0.078	0.138 (0.035 to 0.241)	0.009*
	-Fried food	-0.019 (-0.121 to 0.083)	0.717	-0.154 (-0.285 to -0.022)	0.022*
**MEHHP**	-Light color vegetable	0.023 (-0.054 to 0.100)	0.056	-0.095 (-0.175 to -0.015)	0.020*
	-Fresh fruits	0.144 (0.063 to 0.225)	0.000*	0.092 (0.007 to 0.177)	0.034*
	-Eggs	-0.066 (-0.141 to 0.009)	0.085	-0.091 (-0.168 to -0.015)	0.020*
	-Freshwater fish	0.066 (-0.013 to 0.145)	0.100	0.119 (0.034 to 0.203)	0.006*
	-Lactic acid beverages	0.030 (-0.033 to 0.093)	0.352	-0.068 (-0.131 to -0.004)	0.038*
**MEOHP**	-Light color vegetable	0.021 (-0.049 to 0.091)	0.556	-0.090 (-0.162 to -0.018)	0.014*
	-Fresh fruits	0.145 (0.071 to 0.219)	0.000*	0.096 (0.020 to 0.173)	0.013*
	-Eggs	-0.060 (-0.129 to 0.008)	0.085	-0.092 (-0.161 to -0.024)	0.008*
	-Freshwater fish	0.076 (0.003 to 0.148)	0.040*	0.125 (0.049 to 0.101)	0.001*
	-Soft drinks	0.135 (0.066 to 0.203)	0.000*	0.098 (0.025 to 0.171)	0.009*
	-Lactic acid beverages	0.025 (-0.032 to 0.083)	0.386	-0.067 (-0.124 to -0.009)	0.023*
**∑MEHP** ^**b**^	-Light color vegetable	0.031 (-0.042 to 0.104)	0.408	-0.085 (-0.160 to -0.010)	0.026*
	-Fresh fruits	0.145 (0.069 to 0.222)	0.000*	0.091 (0.012 to 0.171)	0.024*
	-Eggs	-0.069 (-0.140 to 0.003)	0.059	-0.101 (-0.172 to -0.030)	0.006*
	-Freshwater fish	0.073 (-0.002 to 0.148)	0.055	0.129 (0.050 to 0.208)	0.001*
	-Soft drinks	0.132 (0.061 to 0.204)	0.000*	0.088 (0.012 to 0.164)	0.023*
	-Lactic acid beverages	0.030 (-0.030 to 0.090)	0.324	-0.069 (-0.129 to -0.009)	0.023*

*p < 0.05.

^a^Adjusted for age, gender, BMI, parents’ average education level, parents’ smoking condition, parents’ alcohol consumption, guardians who fill in the parental questionnaire, and the other 24 types of food intake.

^b^sum of MEHP, MEHHP and MEOHP, as DEHP-related metabolites.

## Discussions and conclusions

Our study found significant associations between consumption of some types of daily food and phthalate metabolite levels in urine, indicating that dietary intake might be an important source of phthalate exposure among Chinese schoolchildren.

The results showed that metabolites of DEHP were the dominant phthalate contaminators in food, which was in line with Wormuth’s and Chen’s findings in European and Chinese general population [22, 23]. Previous studies mentioned that meat, poultry, and cream-based dairy products were the most frequently reported food with high concentrations of phthalates [9, 16, 18, 24]. However, no significant association was found with these foods in our study and the inconsistency might be attributed to different food cooking methods, e.g. in Chinese dishes, meat may lose those hidden contaminators by being cooked in a long time with high temperature. We found similar results in the relations of di-n-butyl phthalate (DBP) and DEHP metabolites levels with fish and fruits consumption, which was in line with the national studies conducted in the United States [6, 9]; whereas eggs and vegetables showed negative associations with all DEHP metabolites (MEHP, MEHHP, MEOHP and ∑MEHP), which was inconsistent with previous results stating that DEHP had a high concentration in eggs and elevated phthalate concentrations in vivo [9, 18]. Positive associations were found between soft drinks and the level of DEHP secondary metabolites, indicating that soft drinks might be another source of phthalate exposure possibly due to the plastic containers [25]. Those tainted soft drinks could have big impact on children and teenagers considering the popularity of these beverages among the youth. As Serrano et al. discussed in a literature review [24], eggs, fruits, vegetables and beverages were found to contain low concentrations of phthalates and their weak effects might possibly be reduced or reversed by unexpected sources of exposure from ambient environment. Our study showed some inconsistent results with previous researches. The difference might be also due to the different dietary patterns among Chinese children who have less consumption of the foods containing fat such as cream, butter and cheese that might be a major dietary resource of lipophilic phthalates, compared to children in western countries,. On the other hand, Chinese prepared foods depend very much on flavorings and sauces while cooking, and food flavoring, such as salt, would easily contaminate food with phthalate-containing additives [26]. In this study, we only focused on schoolchildren and the results were generally consistent with or only slightly different to the results from the adults [6].

Results in our study indicated that DBP and DEHP were the main phthalate contaminators via diet compared to others. The six phthalate metabolites in our study presented different associations with different food types due to their diverse chemical properties that led to various industrial applications. However, food can be contaminated in many different pathways and the knowledge about the migration of phthalates through diet remains unclear. The general conjecture of the way how food is tainted by phthalates may be attributed to plastics used during processing, packaging, storage and transport of food [16, 27, 28]. The way of phthalate exposure could also be the intake of pharmaceuticals, herbal preparations and nutritional supplements with phthalate plasticizers like DBP and di-ethyl phthalate (DEP) in their coatings [8]. And the detected associations might partially come from other inevitable exposure sources such as air and dermal uptake, which was hard to control in observational studies [22, 23].

In a word, diet is an important source of phthalate exposures among the school-age children in Shanghai, China, and rigorous investigation on exposure route is warranted. Furthermore, studies on exposure to phthalates need thorough considerations about all possible ways with comprehensive investigation of individual behaviors and living habits, regarding the ubiquitous nature of phthalate in environment.

## Abbreviations

FFQ: Food frequency questionnaire
MBP: Mono-butyl phthalate
MMP: Mono-methyl phthalate
MEP: Mono-ethyl phthalate
MEHP: Mono (2-ethylhexyl) phthalate
MEHHP: Mono (2-ethyl-5-hydroxyhexyl) phthalate
MEOHP: Mono (2-ethyl-5-oxohexyl) phthalate
HPLC: High-performance liquid chromatography
LOD: Limit of detection
DEHP: Di-(2-ethylhexyl) phthalate
DBP: Di-n-butyl phthalate
DEP: Di-ethyl phthalate.
